# Pregnant during the COVID-19 pandemic: Knowledge, concerns, attitudes and practices of Pakistani women

**DOI:** 10.18332/ejm/142818

**Published:** 2021-11-25

**Authors:** Rubina Izhar, Samia Husain, Muhammad A. Tahir, Sonia Husain, Saba Hussain

**Affiliations:** 1 Department of Obstetrics and Gynecology, Aziz Medical Center, Karachi, Pakistan; 2 Department of Obstetrics and Gynecology, Karachi Medical and Dental College, Karachi, Pakistan; 3 Abbasi Shaheed Hospital, Karachi, Pakistan; 4 Department of Obstetrics and Gynecology, Aga Khan Hospital for Women Karimabad, Karachi, Pakistan; 5 Department of Obstetrics and Gynecology, Dow Medical College, Dow University of Health Sciences, Karachi, Pakistan

**Keywords:** COVID-19, pregnancy, knowledge, attitudes, practices, Pakistan

## Abstract

**INTRODUCTION:**

Data regarding pregnancy and related outcomes with COVID-19 are inconsistent, which leads to difficulties in counselling pregnant women. This brings uncertainty to pregnant women regarding mode of birth, transmission and issues that may occur in case they contract the disease. We conducted this study to assess the knowledge about COVID-19 risk during pregnancy and childbirth and to assess the concerns, attitudes, and practices of pregnant women during the pandemic.

**METHODS:**

A cross-sectional survey was carried out among 376 consenting pregnant women attending antenatal clinics in Karachi, Pakistan, between 1 July and 16 July 2020, using pretested questionnaires.

**RESULTS:**

A total of 376 pregnant women participated in the survey. Participants had inadequate knowledge about COVID-19 risk during pregnancy, including potential vertical transmission (58.5%), preferred route of delivery (52%), safety of breastfeeding (50%), birth defects (44.7%), rooming in and skin-to-skin contact (58.5%). The majority (85.1%) had a high concern score, and negative attitude (62.8%). Only 43.6% said that they would not hide their symptoms, while 37.2% stated that they will deliver at hospital if they tested positive. Only 30.9% of respondents had good practices. During visits, 39.4% maintained social distancing and sanitized hands while only 37.2% said that they did not bring more than one person with them.

**CONCLUSIONS:**

The study population had inadequate knowledge, negative attitude, and poor practices regarding pregnancy during the COVID-19 pandemic. More than half of the respondents thought that concealing symptoms and delivering at home would be better. Awareness programs are urgently needed.

## INTRODUCTION

Infection from the coronavirus SARS-CoV-2 (COVID-19) was declared a pandemic on 11 March 2020^1^. Since then it has spread to 213 countries. Pakistan has been severely affected, with over 261917 cases and over 5522 deaths^2^. COVID-19 is a novel disease, therefore data on its the clinical course are sparse. Furthermore, its effects on pregnancy are not well known^3^.

Women generally are more likely to display higher levels of psychological distress after natural calamities^4^. Studies have shown high levels of anxiety and fear in pregnant women due to COVID-19^5^. Pregnant women have started showing a greater amount of concern about COVID-19^6^. They miss appointments and frequently show concern about transmission to their baby^7^.

Data regarding pregnancy outcomes are inconsistent, which leads to difficulties in counselling pregnant women. This brings uncertainty to pregnant women regarding mode of birth, transmission, and issues that may occur with breastfeeding, in case they contract the disease^7^. Although reports have been published on the knowledge, attitudes and practices of the general populations regarding COVID-19, data on pregnant women’s perception of the effects of COVID-19 on pregnancy remain scarce^8^.

Counselling plays a major role in allaying fears and helps women cope with pregnancy-associated concerns. Proper counselling cannot be instituted if the providers are unaware of the concerns and current knowledge of their patient. We undertook this study to assess the pregnant women’s knowledge about pregnancy in the pandemic and their attitude, concerns and practices regarding COVID-19^9^.

## METHODS

### Study design

The present study is a multi-facility cross sectional survey conducted from 1 July 2020 to 16 July 2020 on pregnant women attending outpatient clinics of a public sector hospital, a private maternity home, and a private practice.

### Study settings

The study was carried out in the outpatient clinics of obstetrics and gynecology of three hospitals in Karachi. Karachi is the biggest city of Pakistan and has a population of 20 million^10^. Abbasi Shaheed hospital is the third largest tertiary care public sector hospital, Aziz medical center is a private secondary care facility that receives patients irrespective of socioeconomic status while the private practice is located in an area of the city which is frequented by the privileged of the city. The delivery rate for Abbasi Shaheed Hospital is 3600 per year, and around 100 women come for antenatal care each day. The antenatal clinic is held from 9 a.m. to 1 p.m., Monday through Saturday. These clinics are run by consultant obstetricians with their teams of resident doctors and are assisted by nurses. Aziz medical center has a delivery rate of 1000 per year and 40 women attend for antenatal care during each clinic. The clinic is run by two consultants who are assisted by office assistants and nurses. The clinic is held every Tuesday, Thursday and Sunday. The private practice has a delivery rate of 250 per year and 25 women attend for antenatal care in each clinic. The clinic is held twice a week. The clinic is run independently by one consultant obstetrician.

### Sample size

We calculated the sample size using EpiCalc-2000. Our calculation was based on the following assumption: the proportion of good knowledge would be 50%, level of confidence 95% and precision 5%. The sample size was calculated to be 384, and the sample size was increased by 10%, to allow for losses.

### Study instrument

Data were collected using a questionnaire. The questions were designed based on a literature search and frequently asked questions by women during counselling sessions. All questions were discussed with the expert committee that had two obstetricians, one pediatrician, one midwife, a translator, and a psychiatrist. Questions were written in English then translated into Urdu. The questionnaire was then back translated into English. The questionnaire was checked for content and relevance by the authors. We pretested the instrument on 20 pregnant women who were not included in the final analysis. That pretesting showed that the internal consistency of the knowledge (Cronbach’s alpha=0.888), concern (Cronbach’s alpha=0.778), attitude (Cronbach’s alpha=0.678) and practice (Cronbach’s alpha=0.824) sections were good. Data were collected using a questionnaire which was divided into 4 sections: 1) section on demographic and pregnancy related characteristics; 2) knowledge section which had 10 questions covering risk of coronavirus in pregnancy, mode of delivery, breastfeeding and effects on baby, with responses of ‘yes’, ‘no’, or ‘don't know’. A correct response was given 2 points and incorrect response or ‘don’t know’ received 0 points; 3) attitude section had 5 questions that assessed attitude to healthcare in times of COVID-19 (five items), with response to each attitude question was recorded as either ‘yes’ (2 points), ‘maybe’ (1 point) and ‘no’ (0 points); 4) concern section had 5 questions that assessed concerns regarding pregnancy during COVID-19 pandemic, with response to each concern item was recorded as follows: ‘yes’ (2 points) and ‘no’ (1 point); and 5) practice section had 6 questions addressing the woman’s practices during pregnancy, with each question having two responses, ‘yes’ (1 point) and ‘no’ (0 points).

### Study population

Pregnant women, with no symptoms of coronavirus were approached for the survey by the authors. Nonprobability consecutive sampling technique was used. Women attending an outpatient department due to suspected or diagnosed labor and women with obstetric emergencies were excluded. We also excluded women with known psychiatric disorders. The women were approached before their antenatal visit in the waiting area. Women were interviewed by the authors in the clinics of obstetrics and gynecology who were trained to administer the questionnaire. Both the participant and the interviewer wore facemasks during the interview. Translation into native languages was done in cases where the respondents had lower levels of education.

### Data collection and statistical analysis

After obtaining informed consent, we briefed each participant about the purpose of study. Anonymity was ensured and no particulars that could identify the participant were collected. After the data collection, each woman was given accurate and up-to-date information on all aspects of pregnancy and COVID-19. They were encouraged to ask questions and all their misconceptions were cleared. They were also provided the contact numbers of the authors and information leaflets.

Knowledge score was calculated for the 10 knowledge questions as: 1–12 inadequate and ≥13 adequate. Attitude score was: ≤6 negative and ≥7 positive. Level of concern score was: ≤6 low level and ≥7 high.

Practice score was also calculated for practices regarding pregnancy: 1–4 poor practices and ≥5 good.

Statistical Package for Social Science (SPSS) software version 23 was used to analyze data. Descriptive statistical analysis was used to describe items included in the survey. Numbers and mean percentages were used to describe the categorical data. Odds ratios (ORs) with 95% confidence intervals (CIs) were calculated for variables of interest. A p<0.05 was considered statistically significant.

## RESULTS

We approached 466 women to participate and 37 refused participation. Therefore, 429 were included. Of these 429, we excluded 43 due to missing responses; the response rate was 90%. Only those questionnaires that had no missing responses were included in the final analysis. More than two-thirds (n=264; 70%) were aged <30 years and were in their second (39.4%) or third (39.4%) trimester of pregnancy. Table 1 summarizes sociodemographic and pregnancy related characteristics of the study population and scores obtained during the survey.

**Table 1:** Sociodemographic, pregnancy characteristics of study population and scores obtained during the survey (N=376)

*Characteristics*	*n (%)*
**Age** (years)	
19–24	144 (38.3)
25–29	120 (31.9)
30–34	64 (17.0)
35–39	28 (7.4)
≥40	20 (5.3)
**Education level**	
Graduate	80 (21.3)
Inter	88 (23.4)
Matric	108 (28.7)
Not formally educated	100 (26.6)
**Monthly income** (PKR)*	
15000–50000	168 (44.7)
>50000	120 (31.9)
<15000	88 (23.4)
**Employment status**	
Employed	140 (37.2)
Unemployed	236 (62.8)
**Residential area**	
Rural	108 (28.7)
Urban	268 (71.3)
**Source of information on COVID-19**	
Doctors/nurses/midwives	81 (21.5)
Social media	106 (28.2)
Newspaper/news channels	102 (27.1)
Websites (WHO, CDC, Pakistani)	87 (23.1)
**Place of induction of participant into the study**	
Public sector hospital	128 (34)
Private secondary care	162 (43.1)
Private practice	86 (22.9)
**Gestational age** (weeks)	
<13	80 (21.3)
13–26	148 (39.4)
>26	148 (39.4)
**Parity**	
0	51 (13.6)
1	145 (38.6)
2	108 (28.7)
3	40 (10.6)
>3	32 (8.5)
**Scores,** mean±SD	
Knowledge	11.29±6.86
Concern	8.30±1.482
Attitude	5.56±3.433
Practices	3.04±2.13
**Knowledge**	
Adequate	148 (39.4)
Inadequate	228 (60.6)
**Concern**	
High	320 (85.1)
Low	56 (14.9)
**Attitude**	
Negative	236 (62.8)
Positive	140 (37.2)
**Practices**	
Good	116 (30.9)
Poor	260 (69.1)

SD: standard deviation.

* PKR: 1 US$ about 160 Pakistani rupees. Knowledge score for the 10 knowledge questions was: 1–12 inadequate and ≥13 adequate. Attitude score was: ≤6 negative and ≥7 positive. Concern score was: ≤6 low level and ≥7 high level. Practices during pregnancy score: 1–4 poor and ≥5 good.

Only 39.4% women had adequate knowledge. The knowledge was inadequate regarding vertical transmission to baby (58.5%), mode of delivery (52%), breastfeeding (50%), birth defects (44.7%), rooming in and skin-to-skin contact (58.5%). Table 2 summarizes the knowledge section.

**Table 2:** Knowledge of COVID-19 among pregnant women

*Knowledge of COVID-19 and pregnancy*	Yes*	*Don’t know*	*No*
	*n (%)*	*n (%)*	*n (%)*
K1. Pregnant women are more susceptible to corona virus	284 (75.5)	8 (2.1)	84 (22.3)
K2. If I contract coronavirus after 28 weeks, I may have more complications	268 (71.3)	16 (4.3)	92 (24.5)
K3. There is a slight chance of giving coronavirus to my baby if I have coronavirus during pregnancy or childbirth	220 (58.5)	12 (3.2)	144 (38.3)
K4. If I get coronavirus, I can still deliver normally	196 (52.1)	28 (7.4)	152 (40.4)
K5. I should breastfeed even if I have coronavirus	188 (50.0)	20 (5.3)	168 (44.7)
K6. During breastfeeding my face should be covered in case I get the coronavirus	172 (45.7)	44 (11.7)	160 (42.6)
K7. If I get coronavirus, I may still touch my baby (skin-to-skin contact)	220 (58.5)	16 (4.3)	140 (37.2)
K8. Coronavirus during pregnancy can cause births earlier than the due date	188 (50.0)	24 (6.4)	164 (43.6)
K9. According to current evidence coronavirus does not lead to birth defects	168 (44.7)	32 (8.5)	176 (46.8)
K10. If I get coronavirus I can still be in the same room as my baby (rooming in)	220 (58.5)	16 (4.3)	140 (37.2)

* Correct responses, correct response rate (women who answered all questions correctly) 51.98%.

The majority (85.1%) had a high concern score and negative attitude score 62.8%. Less than one-third (30.9%) of the respondents had good practices. Table 3 summarizes attitudes, concerns and practices of the population.

**Table 3:** Concerns, attitudes, and preventive practices during pregnancy, in the pandemic

*Concerns regarding COVID-19 and pregnancy*	*Yes*	*No*	
	*n (%)*	*n (%)*	
C1. Are you concerned about the coronavirus outbreak?	352 (93.6)	24 (6.4)	
C2. Do you feel more vulnerable/weak during the outbreak because you are pregnant?	208 (55.3)	168 (44.7)	
C3. Do you constantly keep thinking that you may have or already have coronavirus?	192 (51.1)	184 (48.9)	
C4. Do you think that your baby might get infected after it’s born?	256 (68.1)	120 (31.9)	
C5. Do you think that you might get infected or right after delivery?	236 (62.8)	140 (37.2)	
***Attitudes***	***Yes***	***No***	***Maybe***
	***n (%)***	***n (%)***	***n (%)***
A1. I trust the information provided by my doctor	272 (72.3)	100 (26.6)	4 (1.1)
A2. I am sure I will be looked after in the best possible way in case I test positive for coronavirus	188 (50.0)	180 (47.9)	8 (2.1)
A3. I feel supported by my obstetrics team during visits	260 (69.1)	116 (30.9)	0 (0.0)
A4. I will not conceal my symptoms from my doctor in case I am infected	164 (43.6)	200 (53.2)	12 (3.2)
A5. I will deliver at hospital if I become COVID-19 positive	140 (37.2)	216 (57.4)	20 (5.3)
***Practices during pregnancy***	***Yes***	***No***	
	***n (%)***	***n (%)***	
P1. I did not skip any appointments due to coronavirus	224 (59.6)	152 (40.4)	
P2. I take precautions whenever I leave my house (cover my face)	200 (53.2)	176 (46.8)	
P3. I used telehealth during this pandemic	180 (47.9)	196 (52.1)	
P4. I have a 30-day supply of medicines and supplements	256 (68.1)	120 (31.9)	
P5. I maintained social distancing during visits and sanitized hands	148 (39.4)	228 (60.6)	
P6. I did not bring more than one person to my visits	136 (36.2)	240 (63.8)	

Table 4 summarizes predictors of knowledge and good practices and Table 5 summarizes predictors of high concern and positive attitudes. Factors associated with adequate knowledge were: age 30–34 years (AOR=0.17; 95% CI: 0.06–0.50, p<0.001); graduate level education (AOR=6.05; 95% CI: 2.27–16.12, p=0.001); rural residence (AOR=0.12; 95% CI: 0.05–0.31, p<0.001); and having monthly household income >50000 PKR (AOR=0.017; 95% CI: 0.05–0.58, p=0.004).

**Table 4:** Sociodemographic predictors of adequate knowledge and good practices regarding COVID-19 in pregnant women

	*Knowledge scores*				*Practice scores*			
	*Adequate (n=148)*	*Inadequate (n=228)*	*AOR (95% CI)^a^*	*p*	*Good (n=116)*	*Poor (n=260)*	*AOR (95% CI)^a^*	*p*
**Age** (years)								
19–24	60	84	0.348 (0.13–0.93)	0.036*	48	96	0.409 (0.15–1.05)	0.064
25–29	40	80	0.20 (0.07–0.54)	0.002*	16	104	0.11 (0.03–0.28)	0.001*
30–34	20	44	0.17 (0.06–0.50)	0.001*	24	40	0.52 (0.19–1.42)	0.203
≥35 (Ref.)	28	20	1		28	20	1	
**Education level**								
Graduate	56	24	6.05 (2.27–16.12)	0.001*	52	28	1.46 (0.52–4.10)	0.473
Intermediate	24	64	0.69 (0.27–1.70)	0.420	16	72	0.18 (0.06–0.54)	0.002*
Matric	48	60	1.55 (0.66–3.63)	0.303	32	76	0.79 (0.30–2.09)	0.646
Not formally educated (Ref.)	20	80	1		16	84	1	
**Monthly income** (PKR)^b^								
15000–50000	72	96	0.62 (0.23–1.63)	0.334	44	124	7.84 (1.85–33.08)	0.005*
>50000	60	60	0.175 (0.05–0.58)	0.004*	68	52	12.23 (2.80–53.28)	0.001*
<15000 (Ref.)	16	72	1		4	84	1	
**Employment status**								
Employed	80	60	1.93 (0.94–3.94)	0.072	76	64	1.91 (0.89–4.12)	0.096
Unemployed								
(Ref.)	68	168	1		40	196	1	
**Residential area**								
Rural	12	96	0.12 (0.05–0.31)	0.001*	12	96	0.76 (0.29–1.97)	0.583
Urban (Ref.)	136	132	1		104	164	1	

AOR: adjusted odds ratio.

a Variables that showed significant associations on bivariate analysis were used in multivariate logistic regression to identify the independent predictors.

* Statistically significant on multivariate analysis.

b PKR: 1 US$ about 160 Pakistani rupees.

**Table 5:** Sociodemographic predictors of high concerns and positive attitudes of women regarding pregnancy in COVID-19 era

	*Concern scores*				*Attitude scores*			
	*High (n=320)*	*Low (n=56)*	*AOR (95% CI)^a^*	*p*	*Negative (n=236)*	*Positive (n=140)*	*AOR (95% CI)^a^*	*p*
**Age** (years)								
19–24	132	12	8.01 (2.05–31.22)	0.003*	88	56	0.42 (0.16–1.11)	0.082
25–29	104	16	4.49 (1.24–16.23)	0.022*	88	32	0.22 (0.08–0.58)	0.002*
30–34	48	16	0.98 (0.29–3.31)	0.977	48	16	0.107 (0.03–0.30)	0.001*
≥35 (Ref.)	36	12	1		12	36	1	
**Educational level**								
Graduate	76	4	7.08 (1.47–34.14)	0.015*	20	60	5.13 (1.85–14.2)	0.002*
Inter	76	12	2.92 (0.84–10.12)	0.090	56	32	1.036 (0.41–2.65)	0.941
Matric	88	20	0.940 (0.29–2.99)	0.917	96	12	0.35 (0.12–1.02)	0.055
Not formally educated (Ref.)	80	20	1		64	36	1	
**Monthly income** (PKR)^b^								
15000–50000	152	16	2.25 (0.74–6.84)	0.151	136	32	0.97 (0.34–2.75)	0.957
>50000	100	20	0.16 (0.04–0.58)	0.005	44	76	1.33 (0.44–3.98)	0.610
<15000 (Ref.)	68	20	1		56	32	1	
**Employment status**								
Employed	132	8	18.08 (4.77–68.49)	0.001*	60	80	3.47 (1.51–7.94)	0.003*
Unemployed (Ref.)	188	48	1		176	60	1	
**Residential area**								
Rural	84	24	1.47 (0.57–3.78)	0.420	64	44	4.39 (1.86–10.30)	0.001*
Urban (Ref.)	236	32	1		172	96	1	

AOR: adjusted odds ratio.

a Variables that showed significant associations on bivariate analysis were used in multivariate logistic regression to identify the independent predictors.

* Statistically significant on multivariate analysis.

b PKR: 1 US$ about 160 Pakistani rupees.

Factors associated with good practices were: age 25–29 years (AOR=0.11; 95% CI: 0.03–0.28, p<0.001); intermediate education (AOR=0.18; 95% CI: 0.06–0.54, p=0.002); and having monthly income >50000 PKR (AOR=12.23; 95% CI: 2.80–53.28, p=0.001).

Factors associated with high concerns were: age 19–24 years (AOR=8.01; 95% CI: 2.05–31.22, p=0.003); graduate (AOR=7.08; 95% CI: 1.47–34.14, p=0.015); being employed (AOR= 18.08; 95% CI: 4.77–68.49, p=0.001); and having monthly income >50000 PKR (AOR=0.16; 95% CI: 0.04– 0.58, p=0.005).

Factors associated with positive attitudes were: age 30–34 years (AOR=0.17; 95% CI: 0.06–0.50, p<0.001); being employed (AOR=3.47; 95% CI: 1.51–7.94, p=0.003); graduate (AOR=5.13; 95% CI: 1.85–14.2, p=0.002); and rural residence (AOR=4.39; 95% CI: 1.86–10.30, p<0.001).

## DISCUSSION

Knowledge about coronavirus and how it affects pregnancy is of paramount importance because it helps mold positive attitudes, allays fears and concerns and leads to good preventive practices in pregnancy. Our cross-sectional survey shows that participating Pakistani pregnant women had inadequate knowledge about the relationship between pregnancy and COVID-19. Our findings are in agreement with a Turkish study^9^ on pregnant women that also showed restricted knowledge but are in contrast with the Chinese study^11^ where three-quarters of the women had adequate knowledge. However, in that study women gained their information from doctors. Our women gathered information from social media (28.2%) and therefore had lower levels of accurate knowledge. Previous studies on knowledge of COVID-19 specifically were largely promising in general populations^12^ as well as pregnant population^13^ but the same is not true for knowledge about COVID-19 and its effects on pregnancy.

Knowledge on the subject of vertical transmission has evolved tremendously, earlier reports from China were hugely favorable but later reports depicted a theoretical risk and increased prevalence of preterm deliveries was also noticed^14,15^. Most of the respondents knew that pregnancy can make one more susceptible to COVID-19; but the chance of transmitting it to their newborn during childbirth is, at best, slight. It was encouraging to see that women were aware of new updates. Guidance on breastfeeding, rooming in and skin-to-skin contact differs slightly. WHO recommends breastfeeding for all women, if they are fit to do so^16^. The American Academy of Pediatricians^17^ recommends expressed breast milk and Royal College of Obstetricians and Gynecologists also advises against direct breast feeding in cases of severe infection^18^. Our study participants did not have adequate knowledge of breastfeeding and newborn care. Older age, higher education and monthly income and urban residence were predictors of adequate knowledge in our study. COVID-19 is a public health problem, therefore counselling sessions in outpatient and delivery suites should be started to help dispel misconceptions.

Due to lack of concrete and convincing evidence for vertical transmission, women can have vaginal delivery even if they have COVID-19^19^. Knowledge regarding mode of delivery was also inadequate. In our study, only 52% of women thought that expecting mothers who test positive could still have a vaginal delivery. More than half (57.4%) of the women preferred giving birth at home in case they tested positive for COVID-19. Asymptomatic COVID-19 patients have been shown to develop an exacerbation of pneumonia, postpartum. This can prove fatal and lead to an increased morbidity and mortality^20^. Moreover, obstetric emergencies that occur out of hospital and the delays that maybe encountered during transportation may aggravate maternal morbidity and mortality. Delivering outside hospital can therefore be extremely dangerous and directive counselling is necessary in this regard^21^.

Fear of contracting coronavirus during, or just after delivery, is also a huge area of concern. In our study around two-thirds (62.8%) of women thought that they would get infected during or after birth, compared to 35% of women in a Turkish study^9^. Furthermore, 68.1% thought that their baby might get infected after being born. These women need to be informed that fetomaternal outcomes are superior to births out of hospital. If proper infection control and prevention strategies are employed, the chances of having an infection are lower than those of delivering at home where the birth attendant may or may not use a mask and deliver the baby with proper aseptic technique^21^.

Furthermore, only one-third of expecting mothers (38.1%) had a positive attitude, which is in stark contrast to the Turkish study (85%)^9^ and Chinese study (90%)^11^. It was alarming that more than half (53.2%) of the women stated they would hide their symptoms from their attending maternity care provider. This negative attitude can lead to exponential damage in settings where one pregnant woman comes in contact with many caregivers. Unexpected exposure from seemingly asymptomatic women has led to outbreaks in New York. In Pakistan, unlike New York, universal screening has not been implemented due to lack of resources^22^. Moreover, these women may decompensate post-delivery and multidisciplinary care maybe needed. Data from New York has shown that most (70%) of the COVID-19 patients admitted to a labor ward were asymptomatic^23^. Another misconception therefore needs to be dispelled. Women with symptoms are actually at a higher risk and need to deliver at a health facility to ensure proper monitoring. In our study, older age, employment and higher education, were predictors of positive attitude. Therefore young, unemployed women with lower academic achievement are sensitive to the prevalent infodemic and should not be neglected. They need a reliable source of information so that myths are busted and positive attitudes become more prevalent.

Nine out of every ten (93.65%) women in our study were concerned about the outbreak and more than half (55%) felt weak because of pregnancy, during this pandemic. These numbers are similar to those reported from Turkey, where 80% women felt concerned and 52% felt vulnerable^9^. A recent analysis from Italy^5^ showed that negative emotional constructs were more common after the pandemic was declared. Psychological support is therefore crucial for the pregnant population. A recent analysis has reported high psychological distress and anxiety in pregnant women during COVID-19^24^.

Four out of every ten (40.4%) women skipped appointments due to the coronavirus; the attendance at our clinics fell during the pandemic, which is similar to the trend in China^7^. Telehealth services were also used during the pandemic in many countries including Pakistan^25^. Around half of our women (47.9%) used some form of telehealth during their pregnancy. The preventive practices during antenatal visits were alarming; around two-thirds neither sanitized their hands nor maintained social distancing during their antenatal visits. Pakistan is a densely populated country and social distancing may not be practical in outpatient areas. Recently WHO has urged the use of facemasks in these settings^26^, but our women did not cover their faces when leaving for places where they anticipated crowds. Pakistan has a high maternal mortality ratio (142.5 per 100000 live births) and infection in this susceptible population can be reduced if preventive measures are adopted^27^. In our study women with high household income and higher education had good practices. Preventive measures for COVID-19 are simple; campaigns that stress the importance of social distancing and mask use should be commenced in outpatient clinics.

We recommend that the following steps should be taken to combat the infodemic. The government should start media campaigns to disseminate updated information on COVID-19 and pregnancy. Hospital administrations should paste an infographic on safety of childbirth in hospitals and breastfeeding newborns during pandemics. Each pregnant women should receive at least one counselling session during her visits that addresses her concerns on COVID-19 from a midwife, if needed a psychiatric assessment should be done. Finally, at the time of admission to the labor suite each pregnant woman should be counseled on all aspects of breastfeeding and newborn care during the pandemic.

### Strengths and limitations

To our knowledge this is the first study from Pakistan that reports findings on knowledge, attitudes, concerns and practices of pregnant women about pregnancy during the pandemic. It has a large sample size and is a multi-facility design to ensure generalizability of results to the population.

Our study has one major limitation. It is a cross-sectional survey and only pregnant women in Karachi who attended outpatient clinics were included. In addition, we did not employ any measure to assess anxiety or fear. We excluded all women with a history of psychiatric disorder but high level of concern needs to be evaluated further. Despite these limitations it is a useful contribution to guide authorities about the looming threat of inappropriate healthcare utilization.

## CONCLUSIONS

The study population had inadequate knowledge, negative attitude, and poor preventive practices regarding COVID-19. High quality counselling programs to disseminate accurate and up-to-date information are urgently needed.

## Data Availability

The data supporting this research are available from the authors on reasonable request.
